# Supplementary material for: Learning curve and surgical outcome of robotic assisted colorectal surgery with ERAS program
Source: Sci Rep. 2022 Nov 29;12:20566. doi: 10.1038/s41598-022-24665-w (PMC9709162; doi:10.1038/s41598-022-24665-w)
Supplement: Supplementary file 1 — Supplementary Information 1. [file 41598_2022_24665_MOESM1_ESM.zip › LSC Raw data-Ñ[▒K/065/N_ñw╝╨░O▒Kñσ.pdf]

W75 / W75

臺中榮民總醫院
手術室護理記錄

[redacted]

基本資料

- 室別： 二醫手術室-31室，手術科別： CRS，手術類別： 常規，日期： 2020/02/06
- 進入時間： 1330 ； 麻醉開始時間： 1335 ； Time Out時間： 1420 ； 劃刀時間： 1421 ； 手術結束時間： 1800 送出時間： 1805 ；
- 麻醉方式： GE
  診斷： Malignant tumor of sigmoid colon,T1
  手術名稱： Laparoscopic Anterior resection and anastomosis - malignant
  手術局部用藥： 無
  術前預防性抗生素：
  - 抗生素名稱一： Veterin 2GM IVD/由麻醉護理師給予
  - 時間： 1400
  - 上次簽章: 林昱君

  ，追加預防性抗生素：
- 手術醫師： 蔣鋒帆
- 麻醉醫師： VS 賴慧卿
-
  - 刷手護士A ： 吳雅華，時間： 1330-1800
  - 流動護士A ： 林昱君，時間： 1330，接替流動一： 彭馨慧，時間： 1500，接替流動二： 陳昱穎，時間： 1700-1800

皮膚狀況

A-V Shunt： 無
術前： 完整
記錄者： 上次簽章: 林昱君
術後： 完整
記錄者： 陳昱穎

手術準備

手術臥式 單一手術臥式

-
  - 手術臥式： 截石臥式
  - 使用附件： 矽膠枕頭，腳架
  - 皮膚準備： 10%Aq Betadine，Hibiscrub，其它： 2%Chlorhexidine
  - 電燒： 使用電燒: 電極片位置： 其它： 背部，雙極電燒，記錄者： 陳昱穎

止血帶T： 無

植入物

- 植入物名稱/號碼/數量/部位： Ligaclip/20釘/colon
  Ethicon EC60A/貳釘/colon
  surgiwrap/小/壹/in pelvic

，記錄者： 陳昱穎 上次簽章: 彭馨慧

標本送檢

- 細菌培養 無
- 細胞學 無
- 冰凍切片 無
- 病理標本
  組織名稱一： sigmoid colon
  ，
  組織名稱二： IMA root
  ，
  組織名稱三： distal cut end

- 截肢 無

標本交付 標本： 共? 袋 ，標本： 零 盒 ，標本： 零 瓶 ，細胞學： 零 管 ，Culture Tube： 零 管 ，被交付醫師： R 邱俊凱 ，

- 記錄者： 陳昱穎
- 時間： 1800

傷口術後敷料

傷口敷料： 傷口敷料： 無

- 請於圖上標示部位：

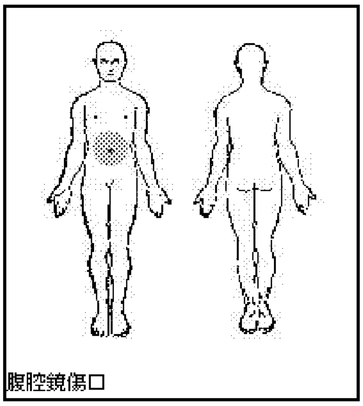

- 傷口敷料： 腹部肆個傷口用Menpor覆蓋固定

導管

導管	天數	導管資訊	插入日期	插入時間	更換日期	拔除日期	拔除時間	拔除原因	預計拔除/更換	預計拔除/更換日期
Penrose Drain(1)	1	號碼:12 mm.; 部位:骨盆腔; 註記:左腹;	2020/02/06	1720						
NG Tube(1)	1	號碼:16 Fr.; 部位:胃;	2020/02/06	1400						
Foley(1)	1	號碼:16 Fr.; 部位:膀胱;	2020/02/06	1400						

用物計數

- 手術中出血量： minimal c.c.
- 手術中尿量： 150 c.c.
-
    - 其它： 無

用物計數： 多切口同時縫合

- 1.器械： 正確 ，2.縫針： 正確 ，3.刀片： 正確 ，4.紗布： 正確 ，5.腹部墊： 未使用 ，6.紗球： 未使用 ，7.PEANUTS ： 未使用 ，8.紗條： 未使用 ，9.絲帶： 未使用 ，10.針頭： 未使用 ，11.Vessel loop： 未使用 ，12.Boots： 未使用 ，13.Jelco： 未使用 ，14.棉條： 未使用 ，
    - 第一次：
        - 刷手護士 ： 吳雅華
        - 流動護士： 陳昱穎
        - 時間： 1735

  ,
    - 第二次：
        - 刷手護士 ： 吳雅華
        - 流動護士： 陳昱穎
        - 時間： 1745

  ,
    - 第三次：
        - 刷手護士 ： 吳雅華
        - 流動護士： 陳昱穎
        - 時間： 1755

備註

備註：NIL

- 記錄者：

填寫護理人員 陳昱穎

正式摘要

W75 / W75

doctorRank:B
